# T-Cell Dysfunction as a Limitation of Adoptive Immunotherapy: Current Concepts and Mitigation Strategies

**DOI:** 10.3390/cancers13040598

**Published:** 2021-02-03

**Authors:** Valérie Janelle, Jean-Sébastien Delisle

**Affiliations:** 1 Centre de Recherche de l’Hôpital Maisonneuve-Rosemont, Montréal, QC H1T 2M4, Canada; js.delisle@umontreal.ca; 2 Division of Hematology-Oncology, Hôpital Maisonneuve-Rosemont, Montréal, QC H1T 2M4, Canada; 3 Department of Medicine, Université de Montréal, Montréal, QC H3T 1J4, Canada

**Keywords:** T cells, chimeric antigen receptor, transgenic T-cell receptor, tumor-infiltrating lymphocytes, exhaustion, terminal differentiation, senescence, apoptosis, adoptive cell transfer, immunotherapy

## Abstract

**Simple Summary:**

T cells are immune cells that can be used to target infections or cancers. Adoptive T-cell immunotherapy leverages these properties and/or confers new features to T cells through ex vivo manipulations prior to their use in patients. However, as a “living drug,” the function of these cells can be hampered by several built-in physiological constraints and external factors that limit their efficacy. Manipulating T cells ex vivo can impart dysfunctional features to T cells through repeated stimulations and expansion, but it also offers many opportunities to improve the therapeutic potential of these cells, including emerging interventions to prevent or reverse T-cell dysfunction developing ex vivo or after transfer in patients. This review outlines the various forms of T-cell dysfunction, emphasizes how it affects various types of T-cell immunotherapy approaches, and describes current and anticipated strategies to limit T-cell dysfunction.

**Abstract:**

Over the last decades, cellular immunotherapy has revealed its curative potential. However, inherent physiological characteristics of immune cells can limit the potency of this approach. Best defined in T cells, dysfunction associated with terminal differentiation, exhaustion, senescence, and activation-induced cell death, undermine adoptive cell therapies. In this review, we concentrate on how the multiple mechanisms that articulate the various forms of immune dysfunction impact cellular therapies primarily involving conventional T cells, but also other lymphoid subtypes. The repercussions of immune cell dysfunction across the full life cycle of cell therapy, from the source material, during manufacturing, and after adoptive transfer, are discussed, with an emphasis on strategies used during ex vivo manipulations to limit T-cell dysfunction. Applicable to cellular products prepared from native and unmodified immune cells, as well as genetically engineered therapeutics, the understanding and potential modulation of dysfunctional features are key to the development of improved cellular immunotherapies.

## 1. Introduction

Adoptive cell immunotherapy (ACT) is a promising approach to treat a variety of pathological states, including infections as well as both solid and hematologic cancers. Immune cells in ACT can be harvested from tumor resection/biopsy, from the patient’s own blood, or donated by a fully or partially human leukocyte antigen (HLA)-matched healthy donor. These cells are then injected into the patient after minimal or more extensive ex vivo manipulations. The oldest, and arguably still one of the most effective forms of ACT, is allogeneic hematopoietic cell transplantation, which most often requires only minimal cell handling and primarily leverages immunogenetic disparities between donor and recipient to treat hematopoietic cancers [1]. Such a prosaic form of ACT is associated with several shortcomings, including unpredictable therapeutic effect and immune complications in the form of graft-versus-host disease (GVHD). Increasingly, however, ex vivo procedures are used to both enhance “on-target” effects on cancer or infected cells and minimize immune complications. This review emphasizes these latter forms of immunotherapy that hinge on advanced ex vivo cellular manipulation procedures. Genetic cellular engineering has also been implemented to achieve optimal cell targeting and minimize off-target effects. In most cases, the manufacturing of therapeutic T-cell products requires T-cell stimulation and expansion, which may be conducive to the acquisition of dysfunctional features [2]. Similarly, continuous and/or chronic antigen encounters after patient infusion often further accentuate T-cell dysfunctionality. Thus, understanding the physiology behind the various T-cell dysfunctional states is fundamental to the design of optimal ACT protocols. 

T-cell dysfunction is not inherently good nor bad, but must be considered as a central aspect of T-cell physiology. In the natural course of an immune response, a high number of T cells are rapidly generated to eliminate the foreign (most often microbial) antigens. After resolution of the threat, a contraction in the total number of T cells limits the risks associated with a sustained inflammatory response and restores homeostasis. Some lymphocytes are differentiated into long-term memory cells to protect the organism against future exposure to the same microorganism. During this process, T-cell differentiation is governed by the strength as well as the duration of the stimulation received by the T cell [3]. The T-cell receptor (TCR) complex, composed of TCRα/β chains and responsible for antigenic recognition, co-operate with a CD3 molecule responsible for transducing the activation signal through its immunoreceptor tyrosine-based activation motif (ITAM)-containing cytoplasmic tail [4]. Phosphorylation of these ITAMs by protein tyrosine kinases then allows other molecules to interact with the TCR complex [5]. Engagement of the TCR, aided by co-stimulatory molecules such as CD28 at the immunological synapse, then activates a wide range of intracellular pathways, including activator protein 1 (AP-1), nuclear factor of activated T-cells (NFAT), nuclear factor-kappa B (NF-κB), and mammalian target of rapamycin (mTOR) [6]. Although triggering of the TCR is a prerequisite for T-cell activation and differentiation, a sustained stimulation can lead to the loss of T-cell functions. In several settings, this is part of homeostatic processes that not only contribute to limiting systemic inflammation, but also protect the T cells against their own demise. Thus, dysfunctional T-cell states result from the sum of physiological countermeasures aimed at controlling T-cell responses and preventing hyperactivation. Several of these regulatory mechanisms are intrinsic to the T cells and others depend on extrinsic factors that can contribute to T-cell dysfunction or use T-cell inhibitory mechanisms to limit immune responses. T-cell dysfunction is defined by different transcriptional, phenotypic, and functional features and encompasses several cellular states such as terminal differentiation, exhaustion, senescence, and/or apoptosis [7,8]. Although the therapeutic opportunities potentially associated with the prevention and/or reversal of T-cell dysfunction are highly relevant to all forms of immunotherapies, including those that mobilize the patient’s own T-cell repertoire such as immune checkpoint blockade and bi-specific antibodies or engagers [9,10], this review focuses on the occurrence of T-cell dysfunction in ACT and the emerging strategies in the field to circumvent this problem.

## 2. T-Cell Dysfunction: Multifaceted Extension of T-Cell Physiology

The current use of T cells as therapy benefits from a large body of knowledge of T-cell dysfunction, obtained from both animal models and humans (extensively reviewed in [11,12,13,14]). This section summarizes the different mechanisms and cell states that can be regrouped under the general term of T-cell dysfunction. The extent to which these cellular states overlap or are exclusive still remains a matter of debate.

### 2.1. Terminal Effector Differentiation

**Figure 1:**
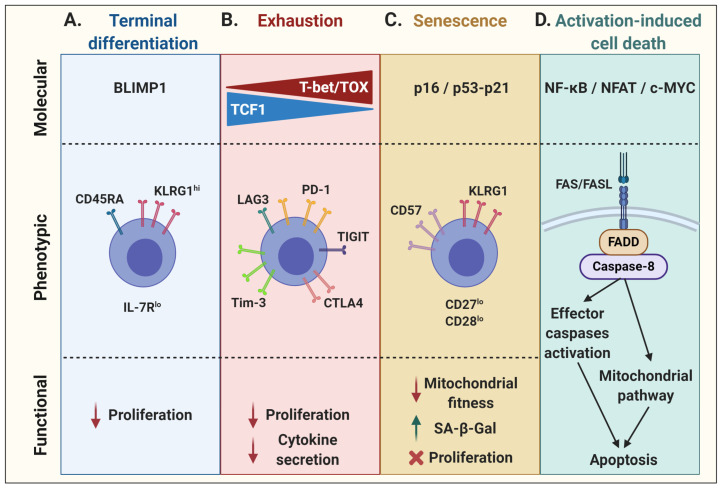
Molecular, phenotypic, and functional characteristics of T-cell dysfunctional states. (**A**) T-cell terminal differentiation, governed by the transcriptional repressor BLIMP1, is phenotypically associated with the re-expression of CD45RA, along with KLRG1, while downregulating IL-7R, leading to an impaired proliferation. (**B**) T-cell exhaustion is regulated by a gradual decrease in TCF1 and concordant increase in T-bet and TOX expression, associated with the upregulation of many inhibitory receptors. In addition to a diminished proliferative capacity, exhausted T cells become hyporesponsive, mainly shown by an impaired cytokine secretion capacity upon stimulation. (**C**) Senescent cells may engage various cell cycle regulators, such as p16 or p21 via p53 expression, to inhibit cell cycle progression. Phenotypically, these cells show upregulation of CD57 and/or KLRG1 at the cell surface, and downregulation of co-stimulatory molecules CD27 and CD28. It is associated with impaired mitochondrial functions, an increase in senescence-associated β-galactosidase (SA-β-Gal) activity, and proliferation arrest. (**D**) Activation-induced cell death requires the activation of a death receptor. Upon TCR stimulation, various transcription factors are induced, such as NF-κB, NFAT, and c-MYC, which can lead to the expression of FASL. FASL may trigger the death receptor FAS on adjacent cells, activating caspase-8 and leading to direct effector caspase activation and/or mitochondrial release of cytochrome c, resulting in apoptosis. This figure was created with BioRender.com.

In physiological conditions, although only a small number of cytotoxic T lymphocytes (CTLs) acquire memory properties following an immune response, a vast majority of effector cells enter a state of terminal effector differentiation. Mainly described in well-characterized mouse models of chronic infection, such as lymphocytic choriomeningitis virus (LCMV), short-lived terminally differentiated CTLs have been phenotypically associated with high expression of the NK cell marker killer cell lectin-like receptor G1 (KLRG1) and low expression of interleukin (IL)-7Rα occurring with the graded expression of the transcription factor T-bet [15,16]. On the one hand, KLRG1 expression impacts proliferative capacity through the decrease of AKT, cyclin D, and cyclin E activity, as well as through an increase in cyclin inhibitor p27 expression [17]. On the other hand, the lack of IL-7Rα prevents T-cell proliferation in response to homeostatic cytokines [18,19,20,21]. Moreover, these terminally differentiated T cells re-express the naïve T-cell marker CD45RA and lose their migration properties in addition to their proliferative capacity [16,22,23,24]. The transcriptional repressor B lymphocyte-induced maturation protein-1 (BLIMP1) may be the most determining factor orchestrating terminal effector differentiation of antigen-specific CD8^+^ T cells [25] (Figure 1A). In addition, it has been shown that hyperglycolysis can induce T-cell terminal differentiation whereas imposition of an oxidative metabolic program may promote T-cell quiescence and loss of effector function [26,27]. At the opposite end of the differentiation spectrum are the so-called early memory T cells (stem cell memory T cells—Tscm, and central memory T cells—Tcm), which have high proliferation potential as well as the capacity to self-renew and persist as long-term memory cells [3]. The skewing of differentiation towards early memory or terminal effector depends on several factors, including the strength and duration of T-cell stimulation as well as multiple intrinsic and extrinsic factors that imprint gene expression programs along with epigenetic marks that strengthen T-cell fates. As described below, modulation of metabolic pathways, cytokine signaling, and epigenetic programming can be used to favor early memory T-cell skewing in lieu of terminal effector T-cell differentiation.

### 2.2. Exhaustion

T-cell exhaustion, driven by chronic TCR signaling, is another evolutionarily conserved process to sustained antigen stimulation aimed at reducing the risks of immunopathology or autoreactivity [12]. Exhausted T cells express multiple inhibitory receptors such as PD1, TIM3, LAG3, CTLA4, and TIGIT, which also leads to a loss of proliferative capacity and effector functions. This state is associated with a gradual increase in the expression of T-bet and a concomitant decrease in TCF1 expression, ultimately rendering exhaustion irreversible [28]. Lately, the role of the calcineurin-dependent transcription factor NFAT and other NFAT-driven transcription factors, such as interferon regulatory factor 4 (IRF4), basic leucine zipper ATF-like transcription factor (BATF), nuclear receptor subfamily 4 group A (NR4A), and thymocyte selection-associated high mobility group box protein (TOX), have been associated with the expression of these checkpoint receptors as well as with the maintenance and survival of exhausted T cells [29,30,31,32,33]. Moreover, TOX expression can further shape the exhaustion transcriptional program and epigenetic landscape of T cells [11]. As a result, these cells have a reduced effector function, mainly shown by a hyporesponsiveness to stimulation and a decrease in cytokine secretion (Figure 1B).

### 2.3. Senescence

While more generally associated with aging, T-cell senescence is typically characterized by cell cycle arrest, activation of the DNA damage response, increased β-galactosidase activity, and dysfunctional mitochondria. These cells nonetheless remain viable and metabolically active [34,35,36]. T-cell senescence is associated with a decrease in the expression of co-stimulatory molecules, such as CD28 and CD27, and an increase in KLRG1 and/or CD57 expression [37,38]. As the T cells divide due to repeated stimulation, they may enter a stage of replicative senescence and lose their proliferation capacity as a result of telomere erosion and a loss of telomerase activity [39,40,41]. Alternatively, a state of “premature” senescence may develop in T cells that sustain DNA damage. In both instances, the expression of cell cycle regulators is upregulated. On one side, the tumor suppressor p53 can activate the cyclin-dependent kinase inhibitor p21, which is responsible for initial cell cycle arrest by inhibiting cyclin-dependent kinase 2 (CDK2), thus preventing S-phase entry. On the other side, upregulation of the cyclin-dependent kinase inhibitor p16 can inhibit the binding of CDK4/6 to cyclin D, restraining this complex to phosphorylate the transcriptional regulator retinoblastoma (RB) protein and preventing cell cycle transition from G1 to the S phase, leading to senescence [42,43,44,45,46,47]. Whereas p16 is mainly associated with cell stress and/or DNA damage, and thus implicated in premature senescence, telomere attrition generally induces the p53–p21 axis (Figure 1C) [42,43,44,45,46]. In addition, telomere-independent, DNA damage-associated cellular senescence has also been linked to p38 mitogen-activated protein kinase (p38MAPK) signaling in activated T cells [48,49].

### 2.4. Activation-Induced Cell Death 

The maintenance of peripheral immune tolerance by activation-induced cell death (AICD) is another key physiological process in T-cell biology [50]. Indeed, TCR stimulation can result in the initiation of apoptosis. Although ligation of TNF-α/TNF receptor 1 and TRAIL/DR4/DR5 have been shown contribute to AICD [51,52], FAS/FASL is the prototype receptor/ligand pair of the extrinsic pathway of apoptosis [53,54]. FASL expression in activated T cells is regulated by several transcription factors [50]. Ensuing TCR stimulation, NF-κB signaling induces the transcription of various target genes such as *FASL* [55]. Along the same lines, NFAT consensus sequences can be found on the *FASL* promoter to regulate its expression, which also requires the co-operation of AP-1 and secretory protein-1 (SP-1) [56]. In addition, FASL expression by T cells can be regulated by c-MYC following its dimerization with its partner molecule MAX to form an active complex for the transcriptional regulation of the *FASL* gene [57]. TGFβ has been shown to control FASL expression by downregulating c-MYC expression, which inhibits T-cell AICD [58]. Upon FAS engagement, a death-inducing signaling complex forms with the adaptor protein FAS-associated death domain (FADD) and procaspase-8, leading to the activation of caspase-8 and subsequent activation of effector caspases, resulting in apoptosis [59]. Activated caspase-8 can further activate a mitochondria-mediated pathway by specific cleavage of the BH3-only B-cell lymphoma-2 (BCL-2) family member BID [60,61]. This converges towards the intrinsic apoptosis pathway, involving other pro-apoptotic Bcl-2 family members, such as BIM, in mitochondria [62,63]. Conversely, T-cell survival mediated by the anti-apoptotic BCL-2 protein was shown to be largely dependent on the IL-7 homeostatic effect [64,65]. As for the other dysfunctional cell states, the nature as well as signal strength of the T-cell stimulation greatly influences cell survival [66,67] (Figure 1D).

## 3. Biology Meets Therapy: T-Cell Dysfunction in Adoptive Cell Therapy 

This section reviews the current ACT approaches and how the notions of T-cell dysfunction integrate in both the manufacturing phase and the post-infusion period. Approaches aimed at mitigating or reversing T-cell dysfunction are also discussed.

### 3.1. Conventional T Cells

Adoptive transfer of ex vivo expanded T cells from the natural repertoire is a promising approach to treat a variety of cancers. However, this requires stimulatory signals to promote in vitro proliferation of T cells that impart a T-cell differentiation program that is susceptible to inducing T-cell dysfunction. Hence, understanding how T-cell dysfunction develops or is programmed by the ex vivo expansion process is imperative. Strategies to promote optimal differentiation and functionality are susceptible to promoting better function and persistence of the transferred cells.

Allogenic hematopoietic cell transplantation is not only the first established form of ACT, it has also served as an ideal setting to advance the field. One of the greatest successes of ACT in the wake of allogeneic hematopoietic cell transplantation is the treatment of immunosuppression-associated opportunistic virus reactivations. Antiviral T cells expanded ex vivo have proven to be highly effective in controlling such viral reactivations in both hematopoietic and organ transplantation [68,69,70,71,72]. Over the years, manufacture duration for these virus-specific T-cell products has gone from three months to around 10 days [73]. It is also possible to directly enrich virus-specific T cells from a healthy donor apheresis product and administer the T cells without further expansion [74]. Most of the published experience with virus-specific T cells reports on the generation of cellular products manufactured from a robust memory repertoire in healthy donors. Such memory cells readily expand in the culture and show limited evidence of T-cell dysfunction at the time of infusion. In hematopoietic cell transplantation, long-term persistence of the transferred cells, thereby establishing long-lasting memory in the recipients, has been shown when the virus-specific T cells are prepared from the original stem cell donor [69]. However, the stimulation and expansion of T cells from naïve repertoire has proven to be more difficult. Although clearly feasible for virus-specific T cells [75,76], the generation and expansion of T cells targeting tumor-associated antigens (TAA) or transplantation antigens from naïve repertoires require more elaborate culture processes [1,2,77,78,79]. 

Alloreactive donor T cells can recognize major histocompatibility complex (MHC)-bound polymorphic peptides derived from the host proteome, known as minor histocompatibility antigens (MiHAs) [80,81,82,83]. Most of the molecularly characterized MiHAs are encoded by autosomal genes that differ between patient and donor secondary to germline-encoded non-synonymous single nucleotide polymorphisms (ns-SNP). Although potent immunogenic antigens, up to five rounds of ex vivo weekly stimulation with antigen-loaded dendritic cells and the presence of interleukin (IL)-2 are often needed to generate high numbers of specific CD8^+^ T cells [2]. However, despite the immunogenic nature of these antigens, protocols often generate dysfunctional cells that highly impede cell functionality and persistence after adoptive transfer [78,79]. Repeated ex vivo antigen exposure in the context of stimulatory cytokines eventually blunts T-cell growth and leads to terminal differentiation and exhaustion marker expression, especially in antigen-specific T cells in the culture [2].

It is now generally accepted that optimal therapeutic effects are achieved when the ex vivo expanded T cells maintain features associated with early memory differentiation (Tscm or Tcm) [3,84]. The clinical outcome might thus depend more on the antigen-specific T-cell early differentiation phenotype, leading to a better ability to proliferate and persist in vivo, rather than on the bulk number of infused cells [85]. As such, careful design of ex vivo culture conditions may promote the acquisition of a favorable differentiation status. Exogenous cytokines, small molecules altering cell signaling, metabolic modulation, and epigenetic modification during the ex vivo priming and expansion phase may confer early memory features [86]. We and others have demonstrated that exogenous exposure to IL-21 can limit terminal differentiation of antigen-specific T cells, which can increase their in vivo persistence and promote phenotypic and functional characteristics associated with long-lived memory T cells [77,87]. The histone deacetylase inhibitor (HDACi) combined with IL-21 can also reprogram differentiated CD8^+^ T cells into central memory-like T cells. This is achieved through an increase in histone H3 acetylation and chromatin accessibility at the *CD28* promoter region. It is then followed by an IL-21-mediated phosphorylation of the signal transducer and activator of transcription 3 (STAT3) binding to the *CD28* region, and a resulting memory-associated transcriptional signature [88]. As the epigenetic landscape of the terminal effector and exhausted T cells is being defined, it is likely that several interventions involving epigenetic modulation are introduced in T-cell manufacturing protocols [89,90]. As such, the inhibition of DNA methylation programs responsible for exhaustion-associated signaling can be a strategy to improve T-cell function [91]. A dominant feature of memory differentiation promoting interventions is the mitigation of activation signals. A brief exposure to the immunosuppressive cytokine transforming factor beta (TGFβ) promotes the Tcm differentiation of ex vivo expanded human T cells [86]. Along the same lines, AKT inhibition has been shown to increase early T-cell memory features [92]. The association of AKT inhibition and early memory T-cell differentiation also highlights how the modulation of pathways associated with cell growth and metabolism are attractive targets for programming T-cell fates. Likewise, limiting glycolysis at the expense of oxidative phosphorylation during ex vivo T-cell expansion has been suggested to improve the fitness of adoptively transferred T cells (reviewed in [26]), and limiting reactive oxygen species metabolism with N-acetylcysteine during T-cell manufacturing can favor early memory T-cell formation [93].

Nonetheless, relying solely on phenotyping may be misleading. The proportion of effector memory or central memory phenotype cells does not necessarily correlate with ex vivo loss of antigen-specific cells or a decline in their functionality [2]. Although repeated antigenic stimulation of MiHA-specific T cells may lead to terminal differentiation, prolonging the expansion phase in the absence of antigenic stimulation can decrease T-cell proliferation despite limited expression of inhibitory receptors and the preservation of polyfunctional cytokine secretion by the remaining antigen-reactive cells. Thus, the analysis of both phenotypic and functional properties of T cells prior to ACT may best inform about the potency of the T-cell product [85]. In addition, it has been shown that a fraction of terminally differentiated melanoma-specific or leukemia-specific CTL clones after ex vivo expansion appears to revert back to a central memory type in vivo after ACT, potentially conferring clinical benefits [77,94].

**Figure 2:**
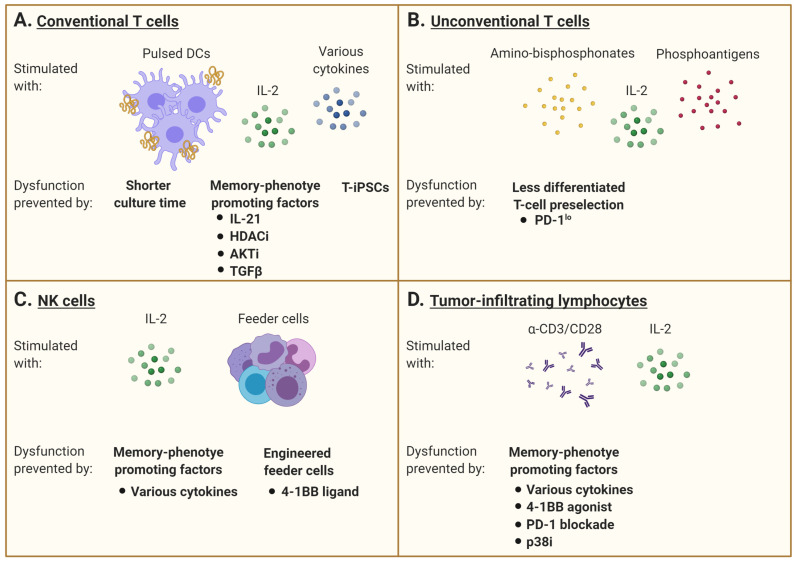
Interventions aiming at limiting T-cell dysfunction for adoptive immunotherapy. (**A**) Conventional αβ T cells are generally stimulated with specific peptides that may be presented by professional antigen-presenting cells such as dendritic cells (DCs) and supported by IL-2 supplementation and/or other cytokines. However, repetitive or chronic antigen stimulation promotes the gradual accumulation of dysfunctional cells over time. Many interventions are under investigation to maintain the cells in a less differentiated state. A shorter culture time allows for the maintenance of a higher percentage of healthy cells, but this may limit the number of cells manufactured. The addition of various factors to promote a memory phenotype differentiation is also evaluated, including the use of IL-21, TGFβ, histone deacetylase (HDAC) inhibitors, or AKT inhibitors. The rejuvenation of dysfunctional T cells by induced pluripotent stem cell technology can also generate less differentiated cells. (**B**) Unconventional γδ T cells are stimulated with amino-bisphosphonates and phosphoantigens, combined with IL-2. The pre-selection of T cells with a favorable phenotype, excluding cells expressing inhibitory receptors such as PD-1, has been investigated to limit the development of dysfunctional features. (**C**) Natural killer (NK) cells are mainly activated with IL-2 and feeder cells. Supplementation with various cytokines or agonists of co-stimulatory molecules (e.g., 4-1BB) can shape their differentiation status and modulate their receptor expression pattern. The engineering of feeder cells as well as the NK cells themselves to express these factors is also underway. (**D**) Tumor-infiltrating lymphocytes are stimulated with anti-CD3 with or without anti-CD28, with IL-2 supplementation. As for conventional T cells, the addition of various cytokines can promote a memory phenotype differentiation. The combination of a 4-1BB agonist and PD-1 blockade can also prevent the development of dysfunction. Inhibition of p38MAPK signaling has further shown to limit development of senescence features. This figure was created with BioRender.com.

As an alternative to limiting the development of dysfunction, some groups have concentrated on cell reprogramming. A terminally differentiated or exhausted T cell may be induced into a pluripotent stem cell (iPSC), which enables re-differentiation into a naïve or central-memory phenotype T cell with the re-expression of CCR7, CD27, and CD28 and no exhaustion markers [95,96,97,98]. An interesting characteristic of iPCSs generated from lymphocytes is their ability to keep the rearranged TCR loci of the parental cells, which remain unchanged during in vitro differentiation [95,97]. As such, antigen-specific CTL clones can be preselected and reprogrammed into iPSC (T-iPSCs) with the Yamanaka transcription factors (Oct3/4, SOX2, KLF4, and c-MYC) [95,96,97,99,100]. These new CTLs then have longer telomeres than the original cells, with higher proliferative and functional capacities [95]. Furthermore, exhausted T cells turned into T-iPSCs are functionally able to respond to antigen-specific stimulation [101]. However, a caveat with the use of iPSC in cell therapy is the risk of only partial re-differentiation and teratoma formation post-transfer (Figure 2A). 

### 3.2. Unconventional T Cells 

γδ TCR-expressing T lymphocytes is another T-cell subtype with effector and regulatory functions and the ability to infiltrate tumors. Adoptive cell therapy with human γδ T cells expressing a Vγ2Vδ2 TCR has shown promise because of their capacity to recognize and kill most types of tumors in an MHC-unrestricted manner [102]. Vγ9Vδ2 T cells are relatively abundant in human blood and can be easily ex vivo expanded in response to amino-bisphosphonates (N-BPs) or phosphoantigens (PAgs). However, culture conditions, timing, and dosage of N-BPs or PAgs, as well as added co-stimulators such as IL-2, may result in different phenotypes and effector cell characteristics [103,104]. Results of in vitro expansion are often highly donor dependent and may also predict the respective in vivo expansion efficacy, which can be additionally restricted in cancer patients. Currently, optimal doses of N-BPs or PAgs as well as IL-2 have not yet been determined for ex vivo expansion. The efficacy of stimulation may depend on drug concentration as well as duration of exposure, which have to be individualized [105]. The role of additional systemic application of N-BPs in the context of adoptive cell transfer strategies also remains elusive. On one side, it has been reported to promote the engraftment of ex vivo-stimulated and adoptively transferred human cells in mice, but on the other side, there are indications that repetitive application of these drugs in vivo induces Vγ9Vδ2 T-cell exhaustion [103]. As an alternative, some groups tried to adoptively transfer PD-1^lo^ Vδ2^+^ T cells to bypass the tumor immunosupressive environment in vivo (Figure 2B) [106].

Other T cells with “innate-like” characteristics can recognize vitamin metabolites, small phosphoantigens, and lipid antigens presented within various highly conserved and non-polymorphic MHC class I-like molecules [107,108,109,110]. One of the best characterized subsets is invariant natural killer T (iNKT) cells, which recognize lipid antigens bound within the antigen-presenting molecule CD1d [111]. These cells utilize the near-germline TCRα rearrangement Vα24-Jα18 combined with a limited TCRβ repertoire and are functionally defined by their ability to respond to galactosylceramide (α-GalCer) when presented by CD1d molecules [107,108,109,110,112,113]. They are potent cytokine secretors that bridge innate and adaptive immunity [109,111]. These cells have thus been investigated as cell transfer therapy products [114,115,116,117,118,119]. However, whether these cells develop dysfunctional features prior, during, and after therapy is still unclear. Nevertheless, since iNKT cells have been shown to limit GVHD [120], there is a growing interest to use them as a platform for cell engineering [121].

The development of ACT using other lymphoid cells is also rapidly expanding. Innate lymphoid cells (ILCs) derive from common lymphoid progenitors in the bone marrow that lack other lineage markers and genetically rearranged antigen receptors. They are defined according to their cytokine production pattern as well as unique transcription factors [122,123,124]. Group 1 ILCs (ILC1s) secrete IFNγ and express the transcription factor T-bet, whereas ILC2s produce IL-5 and IL-13 and require expression of Gata3 [125,126,127,128]. ILC3s generate IL-22 and IL-17 and are defined by the expression of RORγt, as with lymphoid tissue-inducer cells, which also express IL-7Rα [129,130,131]. Since ILCs can respond to many danger signals such as innate immune cells and secrete cytokines such as T cells despite the lack of TCR, different therapeutic approaches aim at targeting these cells in vivo to improve the efficacy of tumor immunotherapies [132]. Although the mechanisms underlying dysfunction in these cell subtypes may not be as well understood as for conventional T cells, several strategies emerge to enhance both the in vitro expansion of these cells and their therapeutic potential.

### 3.3. NK Cells

Natural killer (NK) cells can produce a vast array of cytokines/chemokines and are key players in immune responses against tumors and infected cells. NK cells can also directly regulate T-cell responses as well as modulate antigen-presenting cell activation. In solid tumors, NK cell secretion of CC-chemokine ligand 5 (CCL5), XC-chemokine ligand 1 (XCL1) and XCL2, and IFNγ can promote the recruitment of dendritic cells and further mediate their activation, which have been shown to improve patient outcome [133,134]. However, as with T cells, NK cells can become exhausted, and blockading TIGIT has demonstrated potential in preventing NK-cell dysfunction. Hence, a direct checkpoint blockade in NK cells may result in a more potent tumor-specific T-cell response in an NK cell-dependent manner [135,136].

Activated NK cells also upregulate many receptors, which can be shaped according to culture conditions and media supplementation with cytokines such as IL-2, IL-12, IL-15, IL-18, or IL-21 and Type I IFNs [137,138,139,140,141,142,143,144]. Hence, ex vivo modulation of NK-cell receptor expression has also been extensively investigated to overcome dysfunction. In general, PBMCs are first depleted for CD3^+^ cells and enriched for CD56^+^ cells, then cultured in medium containing IL-2 for up to two weeks [140,145]. This ex vivo stimulation induces NK-cell cytokine secretion, STAT3/AKT signaling, and upregulation of the NKG2D receptor [146]. IL-2 can also enhance NK-cell response to IL-12 by increasing the expression of its receptor [147]. IL-15 supplementation was later used to inhibit activation-induced cell death, and activate the mTOR pathway and stress-activated genes, which confer better anti-tumor capacity [148,149,150]. However, continuous IL-15 signaling has been linked to functional NK-cell exhaustion by decreased fatty acid oxidation [151]. Moreover, it was shown that IL-12-mediated IFNγ production of NK cells requires priming with IL-18, a cytokine also known to enhance IL-15-induced NK-cell proliferation [152,153]. Finally, IL-21 has been used to further increase NK-cell proliferation and effector functions, even though it can trigger apoptosis in vitro [154,155,156,157]. Other studies demonstrated that the duration of NK cell exposure to IL-21 is in fact critical [158,159]. Since NK cells can also express the inhibitory receptor PD-1, it has been found that a blockade of the PD-1/PD-L1 axis can improve NK cell-mediated immunity to tumors, and this response is indispensable for the full therapeutic effect of immunotherapy [160].

Another issue to consider is the NK-cell expansion and functional status from heavily pretreated and thus immunocompromised patients, which are much poorer than for allogeneic NK cells [161]. Other than cytokine supplementation, investigation on feeder cells required for in vitro culture is underway. In a phase I clinical trial (clinicaltrials.gov #NCT02481934), autologous NK cells were activated by an engineered K562 cell-expressed membrane-bound form of IL-15 and 4-1BB ligand [162]. NK cells can also be derived from umbilical cord blood as an allogenic cell source (clinicaltrials.gov #NCT01729091) (Figure 2C).

### 3.4. Tumor-Infiltrating Lymphocytes

Tumor-infiltrating lymphocytes (TIL) are composed of antigen-experienced and “passenger” T cells found at the tumor site. The tumor reactive T cells are subjected to repeated antigen encounters. When harvested for immunotherapy purposes, these cells already exhibit signs of dysfunction that may in fact identify the cancer-reactive T cells [163]. Still, they can be expanded prior to re-infusion into the patient. Although standard protocols use anti-CD3 stimulation with IL-2, generating T cells with a more advanced differentiation state, some groups focused on a cytokine combination cocktail during the expansion phase to increase cell functionality or on the selection of less differentiated TILs among the tumor [23,164,165,166].

TILs found in solid tumors indeed represent a heterogeneous population. It was recently discovered that TILs can be divided into two functionally distinct subsets [167]. The first is the most abundant and is constituted of a clonally related terminally differentiated population that expresses high levels of inhibitory receptors. The other is a TCF1^+^ stem-like CD8^+^ T-cell population, which is suggested to be a major factor in the success or failure to eradicate a tumor depending on their ability to be sufficiently stimulated by an antigen-presenting-cell niche and to continuously produce terminally differentiated CD8^+^ T cells within the tumor [167].

It has also been found that the presence of the integrin αEβ7 (CD103), characteristic of tissue-resident memory T cells (Trm), is positively associated with cytokine production, whereas expression of the transcription factor EOMES is negatively associated with TIL function, suggesting a competition between an antitumor CD103+ Trm-like and an exhaustion program [168]. CD69^+^CD103^+^ Trm cells usually reside in non-lymphoid tissues and function as a first line of defense against secondary infections. In addition to their unique anatomic location, Trm have distinct transcriptional profiles showing the upregulation of inhibitory receptors such as PD-1. However, human Trm have an enhanced capacity for production of certain cytokines and regulatory molecules and a decreased turnover compared to circulating effector memory T cells, suggesting long-term maintenance in situ [169]. Furthermore, it has been demonstrated that the transcription factor BHLHE40 is specifically required for both Trm and TIL development as well as their polyfunctionality by sustaining mitochondrial fitness and a functional epigenetic state. Local PD-1 signaling in the tumor microenvironment inhibits TIL BHLHE40 expression, and BHLHE40 is critical for TIL reinvigoration following anti-PD-L1 blockade [170]. 

Another approach to improve TIL fitness is to target the member of the tumor necrosis factor receptor superfamily T-cell co-stimulatory receptor 4-1BB (CD137). As 4-1BB is frequently present on non-exhausted CD8^+^ TILs, a 4-1BB agonist and a PD-1 blockade demonstrated a synergistic survival benefit in a CD8^+^ T-cell dependent manner. As such, combined treatment decreased TIL exhaustion and improved TIL functionality in a glioblastoma model [171]. Similarly, a PD-1 blockade and 4-1BB stimulation were demonstrated to be an effective strategy to improve pancreatic tumor-reactive TIL yield [172].

Following tumor infiltration, T cells interact with other immune, cancer, and stromal cells within the tumor microenvironment (TME). Cells comprised in this environment may develop premature senescence caused by external factors such as TME metabolic changes or drug and radiation therapy [173]. Senescent cells stay metabolically active but cease to proliferate [174,175]. Another important characteristic of senescent cells is that the expression of many genes changes during senescence and gives rise to what is called the senescence-associated secretory phenotype (SASP) [176]. Senescent cells secrete numerous biologically active factors, including cytokines, chemokines, growth factors, and proteases [177,178]. Because these secreted factors act in autocrine and paracrine manners and have pleiotropic effects on surrounding cells, they may virtually affect any cell type within the tumor microenvironment, including infiltrating T cells. Indeed, tumor-induced senescence in T cells can be reproduced in vitro by briefly incubating cells in conditions of a low tumor-to-T-cell ratio. Furthermore, senescent T lymphocytes become able to suppress the proliferation of normal T cells and promote tumor immune evasion [179]. In anti-CD3 + IL-2 stimulated T cells, the inhibition of p38MAPK signaling proved to be helpful in reversing the senescence phenotype of CD8^+^ T cells by increasing their proliferation and functionality [48,49] (Figure 2D).

### 3.5. T-Cell Receptor (TCR) Transgenic Cells

**Figure 3:**
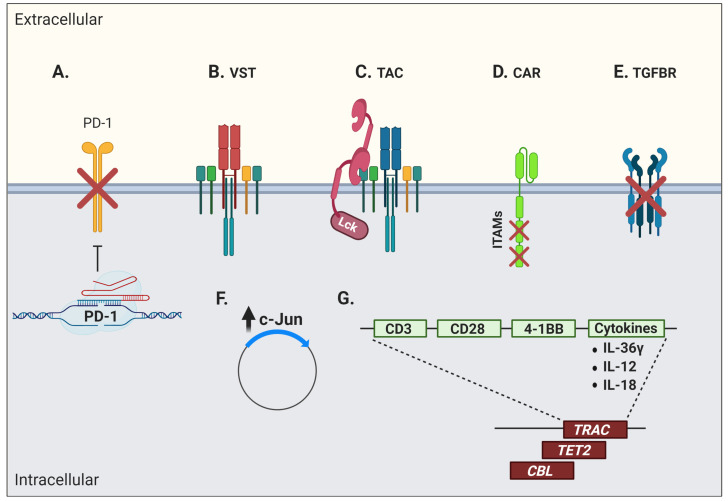
Interventions aiming at limiting T-cell dysfunction in engineered cells for adoptive immunotherapy. Engineered cells comprise transgenic T-cell receptor (TCR) and chimeric/synthetic antigen receptor T cells. (**A**) Genetic ablation of negative co-signaling molecules (e.g., PD-1) may be used to circumvent the effects of T-cell dysfunction. (**B**) Virus-specific T cells (VSTs) have been evaluated as a platform for artificial receptor expression in order to leverage their robust memory differentiation. (**C**) Another strategy to increase modified T-cell fitness is the use of a T-cell antigen coupler (TAC) to avoid tonic signaling and leverage endogenous TCR signaling. (**D**) Advanced engineering of CAR molecules involving the inactivation of two out of three ITAM motifs or (**E**) the blockade of suppressive cytokine signaling, here shown by the overexpression of a dominant negative TGFβ receptor, can also promote better T-cell functions. (**F**) An increase in c-Jun activity has been shown to further limit tonic signaling and T-cell dysfunction. (**G**) Disruption of the endogenous TCRα/β chains by CRISPR/Cas9 or specific integration of the CAR vector into the targeted DNA locus (e.g., TRAC) have shown additional efficacy. This was also true when CAR constructs inadvertently disrupted the *TET2* and *CBL* genes. Other strategies include the enhancement of T-cell function through the production of stimulatory cytokines (e.g., IL-36γ, IL-12, and IL-18) or co-stimulatory molecules (e.g., CD28 and 4-1BB). This figure was created with BioRender.com.

Genetic engineering offers several possibilities, such as conferring new antigenic specificities to T cells and circumventing certain limitations linked to T-cell dysfunction. These approaches provide many advantages, as they minimise culture duration and offer the possibility of directly modulating key signaling pathways within engineered cells to reduce dysfunction. Among these strategies is the introduction of an artificial TCR into antigen-specific conventional T cells. Although it can confer more potent antitumor activity [180], TCR gene transfer is fraught with the risk of inappropriate pairing between exogenous and endogenous TCR chains. Several strategies have thus been used to avoid mispairing that can result in suboptimal activity and novel off-target antigen reactivity, potentially leading to harmful immune side effects [181]. Moreover, the transduced TCR must successfully compete with the endogenous TCR chains to form a ternary complex with the CD3 signaling complex [182]. Hence, the native TCRβ gene, or simultaneous TCRα/β genes, have been knocked out using clustered regularly interspaced short palindromic repeats (CRISPR)/Cas9 technology prior to transduction with a cancer-specific receptor of choice, resulting in a stronger and more polyfunctional response in engineered T cells when tested against target cancer cell lines [183,184]. Remarkably, the combination of CRISPR/Cas9 and TCR transgenic therapy has recently been used to knock out both the endogenous TCR chains and the negative co-signaling receptor PD-1 to redirected T cells bearing a transgenic TCR targeting a TAA from NY-ESO-1 [185] (Figure 3A). 

The introduction of a transgenic TCR into virus-specific T cells (VSTs) to redirect their specificity towards cancer antigens has also been investigated [186,187,188,189,190] (Figure 3B). However, expression of a transgenic TCR often results in the downregulation of the endogenous TCR, which unfortunately leads to a reduced anti-viral reactivity [186,187,188,189,190,191]. This is in part explained by the competition for TCR signaling components by the endogenous and exogenous TCRs, and as possible consequences, one may expect lessened control of viral reactivations post-transplantation and poor capacity of TCR-transgenic VSTs to re-expand in vivo upon viral reactivation or vaccination [190,191]. The addition of CD8αβ to the transgenic TCR vector has been used to rescue endogenous MHC class I-restricted anti-viral TCR function [192,193]. These TCR-transgenic VSTs have a predominant central-memory phenotype and their anti-viral reactivity is preserved together with their anti-tumor function [194]. The insertion of the CD8αβ co-receptor also improved antigen recognition by the TCR/MHC complex, recruitment of the tyrosine kinase Lck to the immune synapse, and proper activation of signaling components for T-cell activation, in addition to allowing CD4^+^ T cells to recognize MHC class I-restricted antigens [195,196]. 

Another strategy to redirect T cells in a TCR-dependent, MHC-independent manner has been the use of a T-cell antigen coupler (TAC) composed of an antigen-binding domain, a TCR-recruitment domain, and a co-receptor domain (Figure 3C) [197]. This design recapitulates the architecture of a TCR complex and engages natural cellular pathways. In addition, these modified cells do not show signs of tonic signaling and display a less differentiated phenotype, which results in a potent T-cell product. These TAC-T cells also showed efficient tumor tissue infiltration at early time points post-ACT and great anti-tumor efficacy in a pre-clinical in vivo model of a solid tumor [197]. Along the same lines, another group fused TCR subunits to an antibody-based binding domain to reprogram T-cell specificity in an HLA-independent manner while still taking advantage of the endogenous TCR signaling. These cells showed tumor cell killing in vivo, although they were less efficient in cytokine production, suggesting some degree of dysfunctionality or impaired activation [198].

### 3.6. Chimeric Antigen Receptor (CAR) T Cells

Engineered T cells for the expression of an artificial receptor emerged in 1989 [199]. This first-generation chimeric antigen receptor (CAR) T cell, composed of a CD3ζ chain containing three ITAMs for TCR-like signal transduction fused with single chain fragment of variable region (scFv) antibody, could support T-cell activation and cytotoxicity, but with very limited persistence and in vivo antitumor efficacy [5,200,201]. Second-generation CARs therefore incorporated the two-signal model of T-cell activation by modifying CAR vectors to include a CD28 or 4-1BB co-stimulatory domain providing signals for T-cell effector function, proliferation, and more importantly, persistence [202,203]. Although complicated by massive activation and cytokine release syndrome, second-generation CD19-targeting CAR T cells rapidly entered routine clinical care. Third-generation CAR T cells further incorporate more than one co-stimulatory domain and other modifications or include the inducible caspase-9 suicide gene system as a “safety switch” to limit on-target, off-tumor toxicities [204]. However, lack of CAR T-cell long-term persistence, poor expansion after ACT, and tumor immune escape remain cardinal limitations of this form of therapy [205]. Hence, several issues pertaining to intrinsic biologic T-cell defaults impact the outcome of CAR-based treatments. Tonic CAR CD3ζ phosphorylation, triggered by antigen-independent clustering of scFv, has been shown to induce early CAR T-cell exhaustion. Moreover, integration of CD28 co-stimulation into the CAR vector seems to increase, whereas 4-1BB co-stimulation limits exhaustion induced by persistent CAR signaling [206]. Indeed, stimulation of CD28/CD3ζ CARs activates faster with larger-magnitude changes in protein phosphorylation, which correlates with an effector T-cell phenotype. In contrast, 4-1BB/CD3ζ CAR T cells preferentially express T-cell memory-associated genes and exhibit sustained antitumor activity against established tumors in vivo [207]. Another way to address the issue of tonic signaling is to calibrate the number of ITAMs on the CD3 moiety. Although the presence of one, two, or three functional ITAMs does not impede in vitro function, a single ITAM-containing CAR can outperform the other forms in vivo (Figure 3D). Remarkably, this modified vector also favors persistence of highly functional CAR T cells, inducing long-lived memory cells with effective anti-tumor properties [208].

Beyond concerns pertaining to T-cell engineering, the quality of the “input” material at the beginning of the manufacturing process impacts clinical outcomes. Autologous CAR T-cell efficacy greatly depends on the functional capacity of patients’ endogenous T cells. Indeed, studies have shown that T-cell fitness diminishes throughout the progression of diseases such as chronic lymphocytic leukemia (CLL), implying impaired proliferative capacity, a dysfunctional phenotype, and a decreased T-cell cytotoxicity, which impact the generation of CAR T cells [209,210,211,212,213,214]. Furthermore, molecular and functional T-cell defects are also acquired by a co-culture of previously healthy T cells with CLL cells [213,215,216]. Among these defects, impairment of mitochondrial biogenesis and fitness, accompanied by reduced glucose transporter 1 (GLUT1) reserves, has been identified, which negatively correlates with the persistence of the transferred CAR T cells and clinical outcome [217]. It was further shown that the frequency of memory T cells, defined by a CD8+CD45RO-CD27+ population, in the pre-manufacturing leukapheresis product was significantly associated with clinical response [218,219]. Similarly, cell features and the magnitude of ex vivo expansion when harvested after a response to induction therapy to manufacture B-cell maturation antigen (BCMA)-specific CAR T cells would be expected to be more clinically effective compared to a leukapheresis product from relapsed/refractory multiple myeloma [220]. Finally, despite the generation of a lesser number of cells, the beneficial effects of reduced culture duration manifests in improved in vitro proliferation and effector function, which directly correlates with improved engraftment and anti-tumor function in vivo, even at a six-fold lower dose [221].

The blockade of immunosuppressive pathways has also been investigated to confer superior functionality in CAR T cells. As such, it was demonstrated that exposure to TGFβ impairs proliferation as well as cytokine production of receptor tyrosine kinase-like orphan receptor 1 (ROR1)-specific CAR T cells co-cultured with ROR1-expressing triple-negative breast cancer cells. Thus, the blocking of TGFβ receptor signaling with a specific kinase inhibitor could promote better anti-tumor function in vitro [222]. TGF-β is abundant in several cancer microenvironments (secreted by the tumor cells themselves, stromal cells, or infiltrating immunosuppressive cells such as myeloid derived suppressor cells—MDSC) and is a prime target for enhancing ACT. However, this is to be undertaken with caution, as TGFβ can also be beneficial for T-cell memory differentiation in certain contexts [223]. After adoptive transfer, the overexpression of a dominant negative TGFβ receptor or hybrid receptors converting an inhibitory signal (such as PD-1) into a stimulatory signal are additional strategies to enhance ACT efficacy (Figure 3E) [224,225]. Along the same lines, genetic ablation of negative co-signaling molecules such as PD-1 in CAR T cells, or CAR T cells secreting anti-PD-1 antibodies, is currently being investigated in clinical trials (clinicaltrials.gov #NCT04213469, #NCT04489862) (Figure 3A).

Studies on CAR metabolic pathway activation revealed that AP-1, a transcription factor composed of dimers of c-Jun and c-Fos, was highly solicited. Its activity is regulated by extracellular signals that can repress or activate its transcription. The formation of the AP-1 complex downstream of the TCR signaling induces IL-2 transcription, among other factors [226,227]. CAR T-cell exhaustion has also been associated with a profound defect in the production of IL-2, along with increased chromatin accessibility of AP-1 transcription factor motifs and overexpression of the basic leucine zipper (bZIP) and IRF transcription factors [33,228,229]. Thus, overexpression of c-Jun rendered CAR T cells resistant to exhaustion, enhanced their expansion potential, increased their functional capacity, and diminished their terminal differentiation [230] (Figure 3F).

Fourth-generation CARs, also called “T cells redirected for antigen-unrestricted cytokine-initiated killing” or TRUCKs, are armored to improve cell fitness by inserting genes coding for other molecules such as cytokines, into the CAR vector [231] (Figure 3G). Interleukin-36γ (IL-36γ), for instance, showed significantly improved CAR T-cell expansion and persistence, and resulted in superior tumor eradication compared to conventional CAR T cells. The enhanced cellular function by IL-36γ was mediated in an autocrine manner. Furthermore, the activation of endogenous antigen-presenting and T cells by IL-36γ promoted a secondary anti-tumor response, which delayed the progression of an antigen-negative tumor challenge [232]. IL-18-armored CAR T cells have also demonstrated enhanced proliferation and persistence in pre-clinical models, in addition to inducing a broadened anti-tumor response through endogenous immune effectors [233,234,235]. Similarly, a CAR T cell secreting the pro-inflammatory cytokine IL-12 demonstrated an improved cytotoxicity and the ability to overcome an immune inhibitory microenvironment in several models, but a clinical trial using IL-12-secreting TILs revealed the high risk of toxicity of this approach if IL-12 secretion is not limited in time and space [236,237]. An attractive strategy to limit cytokine secretion into activated T cells is to use a NFAT-inducible system [238]. 

As seen with conventional T cells, the use of endogenous TCR signaling has been investigated to improve the expansion and function of CAR T cells. As such, virus-specific T cells were modified with a CD19-specific CAR vector and infused into patients without prior cytoreductive chemotherapy. This approach is attractive for two reasons: It leverages the qualities of long-lived memory cells and can use viral reactivation as an adjuvant. In patients with viral reactivation, a striking proliferation of CAR T cells was observed with an associated depletion of CD19-expressing B cells, suggesting that dual TCR and CAR stimulation can potentiate engineered cell expansion [239].

Since CAR constructs are usually generated with the use of viral vectors, integration may result in adverse effects, such as oncogenic transformation or uncontrolled growth, transgene expression, or transcriptional silencing [240]. In a case report of CD19-specific CAR T-cell infusion for CLL treatment, an impressive clinical response was associated with the persistence of one major T-cell clone. It was further shown that the random integration of the CAR vector had disrupted the methylcytosine dioxygenase *TET2* gene, which led to an epigenetic profile consistent with altered T-cell differentiation and a central memory phenotype [241]. In a case of anti-CD22 CAR T-cell therapy to treat B-cell acute lymphoblastic leukemia (ALL), a dominant T-cell clone containing a copy of the vector integrated into the second intron of the E3 ubiquitin-protein ligase *CBL* gene was discovered. A loss of CBL has been associated with a reduction in the threshold for T-cell activation and dependence on co-stimulation [242]. As such, integration of the CAR vector in this region resulted in a dominant-negative effect with the normal CBL (and/or its homolog CBL-B) function, thus contributing to the hyperexpansion in response to a small amount of antigen [243]. Hence, attempts to direct the CAR vector into specific DNA regions are obviously of interest. Indeed, the targeted integration of a CD19-specific CAR vector into the T-cell receptor α constant (*TRAC*) locus resulted in a more physiologic CAR expression, therefore limiting tonic CAR signaling and delaying effector T-cell differentiation and exhaustion [244] (Figure 3G).

Given that iPSCs can be easily amenable to genetic transformations in vitro, T-iPSCs can be genetically modified to augment their applicability, potency, and persistence and offer a great advantage compared to primary cells [245]. Thus, antigen specificity could be assigned to T-iPSCs by means of a chimeric receptor [246]. However, optimization is still needed, as these first iPSC-derived CAR-expressing T cells were still prone to more a terminally differentiated phenotype [246].

### 3.7. Other Engineered Cell Types

Other cell types have also gained interest for CAR engineering. For instance, CAR-modified NK cells show a better safety profile than CAR T cells [247]. Furthermore, their shorter half-life, the smaller array of secreted cytokines limiting the possibility of cytokine release syndrome, their CAR-independent natural killing activity, and their greater potential to be made as an “off-the-shelf” cellular product are among the most attractive features of CAR-NK cells [248,249,250,251]. Nonetheless, late differentiation and exhaustion remain an issue in CAR-NK cell ex vivo expansion protocols. As for CAR T cells, memory-like CAR-NK cells could be promoted when membrane-bound IL-21 was added with feeder cells [252]. Attempts have also been made to increase the lifespan of CAR-NK cells in vivo through an IL-15-armored CAR. A phase I/II study (clinicaltrials.gov #NCT03056339) investigating NK cells engineered with a vector containing an anti-CD19 CAR, IL-15, and an inducible suicide gene revealed a high response rate with an excellent toxicity profile [253]. Lastly, the use of cord blood NK cells as the primary cell source could generate greater numbers of CAR-NK cells, thus potentially reducing overall culture time per patient.

Recently, CAR-macrophages have also entered the field. As professional antigen-presenting cells, these modified cells can be reprogramed to direct their phagocytic activity against cancer cells. CAR-macrophages also show beneficial bystander effects by being able to activate dendritic cells and recruit CD8+ T cells to the tumor site [254].

## 4. Summary and Conclusions

Adoptive cell therapy is a promising approach that can be adapted to target virtually any cancer type or infection in a very dynamic and durable manner. Indeed, T cells as living drugs can provide long-term protection against relapse and can cure diseases that are otherwise incurable. In order to fulfill the promise of ACT, the limitations imposed by the various forms of T-cell dysfunction need to be overcome. These interventions depend on our evolving knowledge of the physiological processes governing immune cell differentiation. Important aspects, such as the plasticity and overlap between dysfunctional states, as well as how these cellular processes progress, remain elusive. The effects of manipulations aimed at these mechanisms may also induce compensatory pathways, making these interventions unpredictable. Hence, research in the physiological mechanisms leading to cell dysfunction must be integrated with translational research in T-cell manufacturing as well as carefully designed clinical trials to reveal the most impactful biological processes to target in order to improve T-cell therapies.

**Figure 4:**
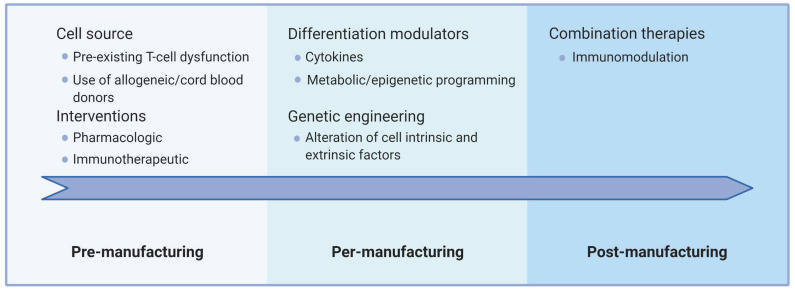
Synthetic representation of concerns and interventions aimed at limiting cell dysfunction before, during, and after manufacturing process. This figure was created with BioRender.com.

Generating such improved immunotherapies requires innovations along two main axes. First, interventions must be directed at the immune cells themselves (intrinsic factors) and/or the multiple cell-extrinsic factors that impact T-cell physiology. Second, improvements may include interventions aimed at the various steps of the ACT procedure, namely, pre-manufacturing (source material), per-manufacturing (cell expansion/differentiation/modification), and post-manufacturing (after adoptive transfer). Figure 4 conceptually summarizes how this can be articulated currently in ACT settings. 

The source material is crucial since dysfunctional cells are already less likely to adequately support strong ex vivo proliferation signals. As such, harvesting less differentiated cells, or using healthy donor cells, facilitates the manufacturing of fit T cells. Whether patient-directed interventions could improve the status of the harvested T cells beyond collecting patients early in their treatment history is an active area of research. For example, Ibrutinib therapy prior to T-cell collection can improve CAR T-cell production in CLL patients [255], highlighting that ACT includes many steps before T-cell injection. Alternative approaches include using healthy allogeneic donors with the caveats imposed by histo-incompatibility (reviewed in [256,257]) and rejuvenation through iPSC generation. The manufacturing phase offers several options that involve the modulation of culture conditions and/or modification of the cells themselves. Several of these have already entered the clinical stage, such as T-cell memory promoting cytokines and genetic ablation of immune checkpoints. It is to be expected that further advances, such as epigenetic programming, will rapidly enter manufacturing protocols. Beyond impacting intrinsic T-cell processes, genetic engineering offers many more opportunities. Adoptively transferred T cells can not only be engineered to resist the immunosuppressive cancer microenvironment (with dominant-negative cytokine receptors, for instance), but can also be modified to enhance trafficking to tumors and alter this microenvironment (through cytokine secretion or secretion of a decoy receptor, among others) [258]. Such “armored” T cells have the potential to reprogram the microenvironment and have an effect reaching beyond the optimization of T-cell function. Similar effects may be achieved through the rationale design of combination therapies that include standard cancer treatments such as radiotherapy, immunological cell death-causing chemotherapy, oncolytic viruses, and other immunotherapeutic approaches [259,260,261,262]. The combination of ACT with a PD-1 blocking antibody is already undergoing clinical evaluation in large trials. However, interference with the homeostatic roles of T-cell dysfunction mechanisms may also be fraught with the danger of triggering unwanted reactivity or even promote further exhaustion or terminal differentiation [263]. Thus, combination therapies or inducible systems to interfere with dysfunctional features more precisely in space and time might be required to fully harness the potential of ACT. It is to be expected that combination therapies involving ACT and advanced engineering cellular protocols will be a major focus of basic and clinical investigation in the next decades.
